# An efficient multiplex approach to CRISPR/Cas9 gene editing in citrus

**DOI:** 10.1186/s13007-024-01274-4

**Published:** 2024-09-28

**Authors:** Cintia H. D. Sagawa, Geoffrey Thomson, Benoit Mermaz, Corina Vernon, Siqi Liu, Yannick Jacob, Vivian F. Irish

**Affiliations:** 1 Department of Molecular, Cellular and Developmental Biology, Yale University, New Haven, CT USA; 2 Present Address: Environmental Sciences Initiative, Advanced Science Research Center, The City University of New York, New York, NY USA

**Keywords:** CRISPR/Cas9, Citrus, Multiplex, Gene editing, Vector design

## Abstract

CRISPR/Cas9*-*mediated gene editing requires high efficiency to be routinely implemented, especially in species which are laborious and slow to transform. This requirement intensifies further when targeting multiple genes simultaneously, which is required for genetic screening or more complex genome engineering. Species in the *Citrus* genus fall into this category. Here we describe a series of experiments with the collective aim of improving multiplex gene editing in the Carrizo citrange cultivar using tRNA-based sgRNA arrays. We evaluate a range of promoters for their efficacy in such experiments and achieve significant improvements by optimizing the expression of both the Cas9 endonuclease and the sgRNA array. In the case of the former we find the *UBQ10* or *RPS5a* promoters from Arabidopsis driving the *zCas9i* endonuclease variant useful for achieving high levels of editing. The choice of promoter expressing the sgRNA array also had a large impact on gene editing efficiency across multiple targets. In this respect Pol III promoters perform especially well, but we also demonstrate that the *UBQ10* and *ES8Z* promoters from Arabidopsis are robust alternatives. Ultimately, this study provides a quantitative insight into CRISPR/Cas9 vector design that has practical application in the simultaneous editing of multiple genes in *Citrus*, and potentially other eudicot plant species.

**Supplementary Information:**

The online version contains supplementary material available at 10.1186/s13007-024-01274-4.

## Introduction

*Citrus* is a genus with globally cultivated fruit crops having substantial economic importance through widespread consumption as whole fruit, juice, or in various ancillary products. Despite their importance as a commodity, developing new *Citrus* varieties through traditional breeding techniques is challenging due to long reproductive life cycles, high heterozygosity, widespread apomixis, frequent polyploidy and variable subgenome paralog numbers in many commercial cultivars [44]. Furthermore, *Citrus* crops are susceptible to a variety of diseases, including Huanglongbing (HLB), a devastating bacterial disease that has reduced citrus production worldwide [50]. Thus, the development of resistant varieties is imperative to sustain the production of *Citrus* crops in the field [13]. Disruption of candidate disease susceptibility genes through the use of CRISPR/Cas9 gene editing is a vital tool in the arsenal of approaches to create disease resistance.

CRISPR/Cas9 as a technique has made targeted genetic modifications in plants feasible and its utilization has the potential to streamline breeding programs by introducing desired traits into elite germplasm in just a few years, as opposed to the decades associated with traditional breeding [66]. This technique has been successfully applied in many crops [46, 53] including *Citrus* [20, 21, 26, 38, 45, 59, 62, 64]. However, these studies are, for the most part, proofs of concept targeting single genes. More effective vectors are required for the simultaneous targeting of multiple loci, or for more sophisticated *Citrus* genome manipulation.

Gene editing with CRISPR/Cas9 requires two components; an endonuclease called Cas9 which creates double-stranded DNA breaks, and a single guide RNA (sgRNA) which forms a complex with the Cas9 protein to confer sequence specificity [6, 22]. Double-stranded DNA breaks can result in an edited sequence if repaired erroneously in a plant cell [61]. In plants such as cultivars of *Citrus*, where transformed explants are regenerated through tissue culture, it is desirable for editing to occur early in the process. This ensures that the majority, if not all, of the plant cells are edited in the T_0_ generation. Obtaining fully edited progeny from the passage of edits through the germline takes many years in *Citrus* due to long reproductive life cycles, which explains the need to achieve high levels of editing in the T_0_ generation to accelerate and facilitate functional analysis of *Citrus* mutants.

Efficient gene editing requires Cas9 and sgRNA to be present together in a plant cell at high concentrations. Thus, in a transgenic context, the promoters used to drive the genes encoding the CRISPR/Cas9 components are of critical importance. Multiple studies have shown that highly penetrant editing in plants can be achieved using promoters that target actively dividing cells in, for example, *Arabidopsis thaliana* (*Arabidopsis*), *Nicotiana*, and *Citrus* [4, 11, 12, 35, 52, 60, 62]. However, the relative utility of these promoters can vary between species and require testing on a case-by-case basis. A complementary approach toward the same end is the introduction of introns into the Cas9 transgene which results in higher gene expression, and thus more protein available to generate edits at targeted loci [15]. Additionally, efficient gene editing requires that the targeted DNA is accessible to the Cas9 complex. This can be aided by exposing the transgenic plants to heat stress, which renders chromatin more accessible to the Cas9-sgRNA complexes [26].

CRISPR/Cas9 gene editing can be used to target multiple independent loci simultaneously, which requires expressing multiple sgRNAs at once. Naturally, this dilutes the cellular pool of unique Cas9 complexes, as the Cas9 protein is shared between all available sgRNAs. This can reduce editing efficiency, so efficiency needs to be sufficiently high to begin with for this approach to be successful. A common method to perform multiplex CRISPR/Cas9 is to express each sgRNA independently, such as through individual gene cassettes regulated by Pol III promoters (commonly isolated from U6 snRNA genes) [31]. Alternatively, all sgRNAs can be organized into a tandem array and expressed as a single transcript from one promoter. This single transcript undergoes post-transcriptional processing to excise individual sgRNAs [20, 47, 51, 57]. This strategy becomes more appealing with an increased number of sgRNAs, as arrays can be commercially synthesized and cloned in a single step. Moreover, in this context, a single Pol II promoter can be used to transcribe the array transcript, providing greater flexibility in regulating the expression of the sgRNAs.

Various strategies have been employed to post-transcriptionally process sgRNA array transcripts [31]. One popular approach is through the use of pre-tRNA sequences interspersed between the sgRNAs in the array. This strategy is effective because ribonucleases P and Z cleave transcribed pre-tRNA sequences at both the 5′ and 3′ ends, respectively [16, 42]. Thus, the inclusion of pre-tRNAs in a sgRNA array transcript results in the release of individual sgRNAs, enabling them to form a complex with Cas9 [65]. Although the use of pre-tRNA sequences in multiplex CRISPR/Cas9 gene editing has been successfully reported in *Citrus* previously, those studies employed multiple sgRNAs targeting a single *PHYTOENE DESATURASE (CsPDS)* gene [19, 20, 45, 47]. However, this strategy has yet to be applied to target multiple *Citrus* genes simultaneously, nor have different promoters been trialed to optimize it.

Here we present our progress in vector design for improving multiplex gene editing in *Citrus.* This is a continuation of our ongoing efforts to advance *Citrus* transformation and gene editing [26, 62]. In these experiments we demonstrate robust expression of the Pol II promoter *ES8Z* (At5g20290) in the *Citrus* rootstock cultivar ‘Carrizo’ citrange (*Citrus sinensis* ‘Washington’ sweet orange X *Poncirus trifoliata*), and developed an efficient multiplex CRISPR/Cas9 gene editing system capable of disrupting at least four genes simultaneously. Additionally, we tested the *ES8Z* promoter alongside previously described promoters in new combinations, and further improved the efficiency with which we could edit *Citrus*. These progressive improvements have enabled the transition of multiplex CRISPR/Cas9 gene editing in *Citrus* from proof-of-concept experiments to more general practical application.

## Material and methods

### Cloning

The creation of the binary vector containing four sgRNAs each under the control of an individual *AtU6-26* promoter was carried out as previously described [60] using the p*YAO*::*hSpCas9* (human codon–optimized *SpCas9*) backbone [62]. A precursor of the p*YAO*::*hSpCas9* for *Citrus* backbone, lacking a terminator following the *hSpCas9* gene, was modified by adding the *rbcS-E9* terminator (*E9*) into the *AscI* restriction site immediately downstream of *hSpCas9*. The *E9* terminator was amplified from pICSL60004 (Addgene plasmid #117519) [2] using primers AddTerm_F and AddTerm_R. A *ES8Z::PacI-MluI::nost* cassette was then cloned into the existing *SpeI* and *SbfI* restriction sites upstream of the p*YAO*:*:hSpCas9*::*E9* cassette. Note the inclusion of the internal *PacI* and *MluI* restriction sites which facilitate the cloning of genic sequences between the regulatory elements. This was done for the *RUBY* gene, amplified from the *35S*::*Ruby* plasmid (Addgene plasmid #160908) [17] using primers getRuby_F and getRuby_R, as well as synthesized arrays of sgRNAs separated by tRNA sequences (*Arabidopsis thaliana*, Glycine, GCC anticodon) (Gene Universal Inc. Newark, DE, USA). The array sequences are listed in Supplementary File 1. To test a broader range of binary vector components we utilized Golden Gate cloning [30, 54] making use of the MoClo toolkit [54], and compatible components. These include both previously published [2, 8, 15, 25] and novel components. The origin and construction of these vectors are detailed in Supplementary Table S2. A list of the plasmids used in this study, as well as references to their maps can be found in Supplementary Table S1. Note that for the purposes of sgRNA design we have used gene identifiers from Hzau_Valencia_v1.0 [58], Citrus_sinensis_v1.1 [56] and Poncirus_trifoliata_v1.3.1 [37] as representative of the Carrizo citrange parental sequences and these are listed in Supplementary Table S3.

### Citrus material and transformation

Seeds of Citrus rootstock cultivar Carrizo citrange (*Citrus sinensis* ‘Washington’ sweet orange X *Poncirus trifoliata* purchased from Lynn Citrus Seed, Inc. in 2021–2023) and ‘Valencia’ sweet orange (*Citrus sinensis* purchased from Lykes Citrus from Lykes Bros, Inc. in 2021–2022) were germinated in vitro (MS with vitamins, 30 g/L sucrose, 2.5 g/L Phytagel, pH 5.8) in the dark at 24 °C for 4 to 6 weeks to promote etiolation, followed by 7–10 days under 16-h-light/8-h-dark photoperiod (40 µmol/m^2^) at 28 °C. The epicotyls of etiolated seedlings were used in transformation as described previously [36] with a few modifications. In brief, 75–100 explants at a time were incubated with *Agrobacterium tumefaciens* strain EHA105 harboring the binary vector in co-cultivation culture (MS basal media with vitamins, 30 mM MES, 1% sucrose, pH 7.0) for 15 min under agitation at room temperature. After draining excess media, dried explants were transferred to plates containing co-cultivation agar media (MS with vitamins, 30 g/L sucrose, 3 mg/L of BA, 0.1 mg/L of NAA, 2.5 g/L of Phytogel, pH 5.8) for a 3-day co-culture in the dark at 24 °C. The explants were then transferred to regeneration medium (MS with 30 g/L sucrose, 1 mg/L BAP, 0.1 mg/L NAA, 250 mg/L cefotaxime, 100 mg/L Ticarcillin, 50 mg/L Kanamycin, 2.5 g/L Phytogel, pH 5.8.) in the dark at 28 °C. Transgenic shoots were identified by screening for GFP signal using a NIGHTSEA system with a royal blue LED light. GFP-positive shoots were transferred to elongation media (MS with vitamins, 30 g/L sucrose, 1 mg/L of BA, 0.1 mg/L of NAA, 250 mg/L of cefotaxime, 100 mg/L Timertin, 50 mg/L Kanamycin, 2.5 g/L Phytogel, pH 5.8) and incubated under previously described light conditions. After elongation, transgenic shoots were cut from the explant and rooted on rooting media (MS with vitamins, 30 g/L sucrose, 0.5 mg/L NAA, 2 mg/L IBA, 2.5 g/L Phytogel, pH 5.8). Well-rooted plants were transferred to MS media for 2–4 weeks and then transferred to soil (Promix) under a plastic dome under a 16-h-light/8-h-dark photoperiod (195 µmol/m^2^), at 28 °C with a relative humidity between 40 and 60%.

### Phenotyping

In addition to GFP screening, the analysis of *RUBY*-transformed explants [17] included screening for the presence of shoots containing red coloration resulting from betalain accumulation. Transformants carrying vectors targeting *CsPDS* underwent an additional screening process for the absence of chlorophyll, manifesting as shoots turning either completely or partially white.

### Betalain quantification

Quantification was performed as previously described with modifications [7]. Three leaf disks of 6 mm diameter from young leaves were placed into a 2 mL tube, followed by the addition of 1 mL of 53 mM phosphate buffer (Na_2_HPO_4_/KH_2_PO_4_, pH = 6.5) and two metal beads. The leaves were homogenized using a TissueLyser at 20.0/s for 2 min. Post homogenization, the leaf homogenate was centrifuged at 10,000 × g for 5 min, and the resulting supernatant was transferred to a new 2 mL tube. This supernatant was centrifuged at 17,000×g for 10 min. The obtained supernatant was diluted by a factor of 3:5 with phosphate buffer. To assess pigment absorbance, measurements were taken at (a) 538 nm, (b) 476 nm, and 600 nm (c) using a 1 mL cuvette to measure betanin, vulgaxanthin-I, and small impurities, respectively [7]. To determine pigment concentration, the following formula was applied: Absorbance of betanin (red pigments) = 1.095 × (a–c). The final concentration was calculated by multiplying the corrected absorbance by the dilution factor and dividing it by the specific absorption at 1% (A_1% = 1120 for betanin).

### DNA extraction and genotyping analysis

Genomic DNA was extracted for Sanger sequencing as previously described [26, 62]. For the whole genome sequencing libraries, DNA was extracted from a pool of leaf disks from plants with the same constructs using the DNeasy Plant Pro kits (Qiagen, Hilden, Germany) and EchoLUTION Plant DNA Kit (BioEcho Life Sciences, GmbH, Köln, Germany) following manufacturer protocols, with the exception of doubling the grinding buffer in the latter case. Pools consisted of leaf tissue from 9 to 146 plants (median 70, see Supplementary Table S5) which had been planted in soil for 2–12 months. The targeted regions were amplified by PCR using GoTaq DNA Polymerase (Promega, WI, USA) following the manufacturer’s protocol and sent to the Yale Keck DNA Sequencing Core facility for Sanger sequencing. DNA sequences were analyzed and the editing level or knockout scores were determined using the Synthego ICE Analysis tool (v3.0) [5] which maps the mutant sequence against a wild-type (not edited) sequence. Primers are listed in Supplementary Table S4. Libraries for pooled DNA whole genome sequencing were prepared following a modified version of the protocol published by Jones et al. [23], including DNA quantification, tagmentation, PCR for enrichment and barcoding, and pool libraries steps. Clean-up and size selection steps were adapted using Illumina’s double-sided size selection and bead clean up (Illumina, CA, USA) to enrich libraries with fragments ranging from 200 to 800 bp and using the HighPrep PCR beads (MAGBio Genomics, MD, USA) for size selection. Libraries were then sequenced on a NovaSeq 6000 (Illumina, CA, USA) operated by the Yale Center for Genome Analysis. FASTQ files were processed using fastp (v0.23.2) [3] (-w 10 -q 20 -l 20) and aligned to a currently unreleased Carrizo citrange genome sequence using BWA mem (v 0.7.17) [27] followed by Abra2 (v2.23) [33]. Alignments were further processed using Samtools (v1.16) [28] and levels of editing assessed using PySam (v0.21) [39]. Analysis was conducted using computing resources provided by the Yale Center for Research Computing. This analysis made use of a currently unreleased Carrizo citrange genome sequence. For the purposes of this publication regions targeted by CRISPR/Cas9 reagents are listed in Supplementary File 2.

### Data availability

Maps of the plasmids used in this study have also been uploaded to GenBank and are listed in Supplementary Table S1. The DNA sequencing data alignment statistics are presented in Supplementary Table S5 and the genotyping is summarized in Supplementary Tables S6 and S7. Raw data has been deposited in the NCBI (Bioproject PRJNA1090434) with SRA identifiers listed in Supplementary Table S8. Sequences of the sgRNA arrays used in this study are present in Supplementary File 1 and genomic regions targeted by CRISPR/Cas9 reagents have been uploaded to GenBank and are listed in Supplementary File 2.

## Results

### Expression Analysis of ES8Z Promoter in Citrus

We sought to investigate a 404 bp ribosomal *ES8Z* (At5g20290) promoter from *Arabidopsis* to test for its activity in *Citrus*. We chose this promoter for its robust expression levels in undifferentiated and dividing tissues of *Arabidopsis*, similar to other ribosomal gene promoters [24, 40, 55]. Such an expression pattern has been previously shown to be advantageous for multiplex CRISPR/Cas9 editing [15, 41, 63].

**Fig. 1:**
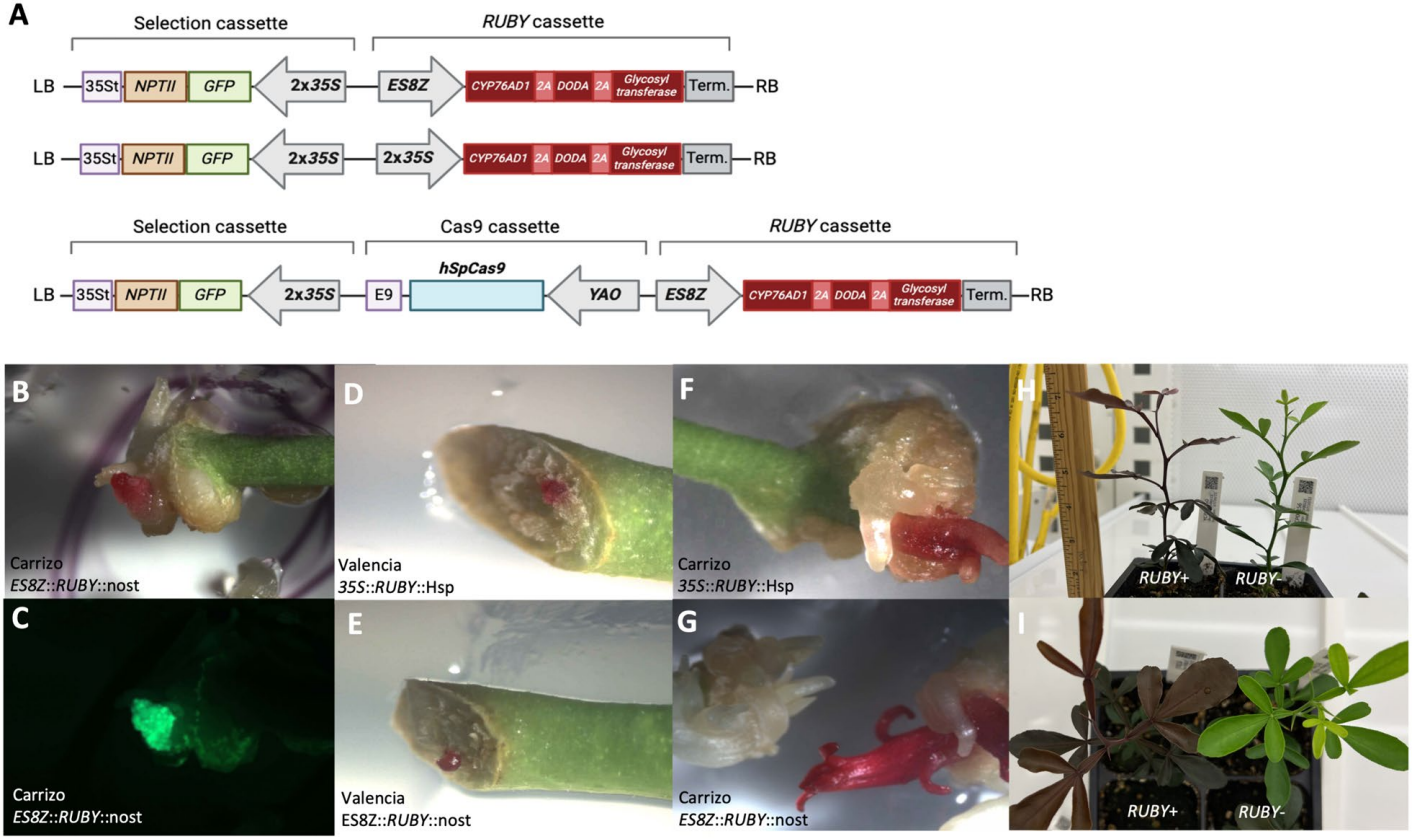
The *ES8Z* promoter drives expression of the *RUBY* reporter in *Citrus*. **A** Comparison of different constructs with the *ES8Z* and *35S* promoters driving the *RUBY* cassette. **B** Carrizo citrange transformed explants with the *ES8Z* promoter expressing *RUBY* and **C** GFP simultaneously. **D**, **E** Valencia explants with *RUBY*-expressing transformed calli. **F**, **G** Carrizo citrange transformed shoots. **H**, **I** Side and top views of *RUBY*-transformed Carrizo citrange plants with no obvious phenotypic differences except for red pigmentation in the *RUBY* + plant

To assess the activity of the *ES8Z* promoter in *Citrus*, we first inserted it upstream of the *RUBY* reporter cassette (Fig. 1A), which produces red betalain from the endogenous supply of tyrosine [17]. We also created a similar construct with *RUBY* driven by the *Cauliflower mosaic virus* (CaMV) *35S* promoter (*35S*) to compare *ES8Z* expression with that of a constitutive promoter (Fig. 1A). In addition, we integrated the *RUBY::ES8Z* cassette into our previously published *Citrus* CRISPR/*Cas9* vector (p*AtYAO*::*hSpCas9*) [62] to mimic the context in which it might be used for multiplex CRISPR/Cas9 editing (Fig. 1A). Both constructs without the Cas9 cassette exhibited successful expression of *RUBY* when transformed into explants derived from two different *Citrus* cultivars: Valencia and Carrizo citrange (Fig. 1B-G). However, only Carrizo citrange explants developed shoots which could be elongated and rooted (Fig. 1F-G). GFP, which is used to identify transformed plants, was expressed simultaneously with *RUBY* in several explants/shoots (Fig. 1B-C), although not all fluorescent tissue necessarily exhibited the red pigmentation.

The vector containing the *ES8Z::RUBY* cassette without the *Cas9* cassette produced 122 transgenic plants (GFP +) from 137 Carrizo citrange explants, a transformation efficiency of 89%. Transformation of the vector containing *ES8Z::RUBY* which included the *Cas9* cassette yielded 35 GFP + transformants, but had a more modest transformation efficiency of 44.3%. Red pigmentation, indicative of *RUBY* expression*,* was observed in 80.3% and 31.4% of these transformants, without and with the Cas9 cassette respectively. Conversely, the vector containing the 2 × *35S*::*RUBY* had a relatively low transformation efficiency of 27.8%, yielding 27 transformants, of which three transgenic plants (11.1% of transformants) exhibited red coloration (Table 1).

**Table 1:** Summary of transformation efficiency and expression of *RUBY* transgene in Carrizo citrange plants transformed with vectors testing the activity of the *ES8Z* promoter

*RUBY* Cassette	Number of Carrizo explants	Number of transformed plants (GFP +)	Transformation efficiency (percentage)	Number of transformed plants expressing *RUBY*	Transformed plants expressing *RUBY* (percentage)
*ES8Z*::*RUBY*	137	122	89.1%	98	80.3%
2 × *35S*::*RUBY*	97	27	27.8%	3	11.1%
*ES8Z*::*RUBY* (*YAO*::*hSpCas9*)	79	35	44.3%	11	31.4%

Transformation efficiency was defined as the number of transgenic plants arising from transformed explants grown on media containing kanamycin as well as the expression of GFP. *RUBY* transgene expression was inferred from red pigmentation of the plant

In the plants where RUBY was expressed, the pigmentation was sustained throughout plant development (Fig. 1H-I). These results highlight the efficacy of the *ES8Z* promoter in driving robust and efficient expression in *Citrus*, particularly in the Carrizo citrange cultivar.

Among the transgenic Carrizo citrange plants, the intensity of the red pigmentation from the *RUBY* reporter varied considerably (Fig. S1A). This variation ranged from intense red pigmentation to the complete absence of red pigmentation, resembling wild-type leaves. The accumulation of betalain was quantified in individual plants over time, revealing a significant reduction after plants reached 10 weeks of age under our growth conditions (Fig. S1B). Furthermore, some transgenic plants exhibited tissue-specific expression patterns, with only new leaves displaying red pigmentation (Fig. S1C), or only roots showing a distinctive pink color (Fig. S1D). These findings likely reflect the variation in transgene activity which arises from variation in transgene integration [48] and is likely attributable to differential gene silencing of the transgenes [1]. However, the reduction in betalain accumulation over time is also consistent with the *ES8Z* promoter being more active in young actively dividing tissues.

### Evaluation of *ES8Z* promoter performance in a multiplex sgRNA system

**Fig. 2:**
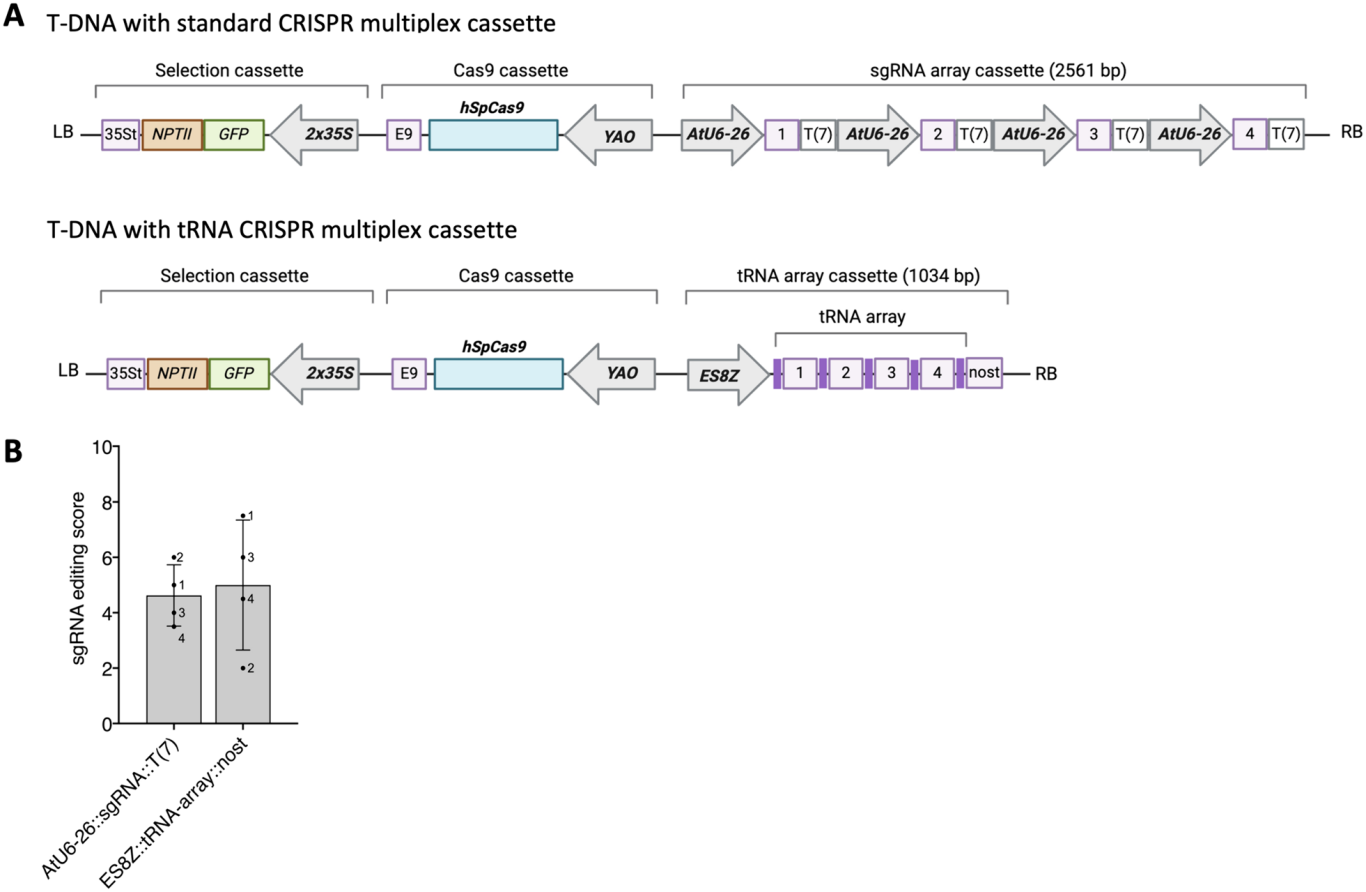
Comparison of multiplex gene editing using individual *AtU6-26* driven sgRNA cassettes relative to a *ES8Z* driven tRNA-based sgRNA array in Carrizo citrange. **A** Schematic representation of T-DNA transformed in this experiment. **B** Gene editing scores (1—low; 10—high) from whole genome sequencing of pooled leaf tissue (46–89 plants) from the two populations of transgenic plants. The bars show the average of the sgRNA editing score for each vector, error bars refer to the standard deviation and numbers on the bars refer to the position of the sgRNA on the vector

We next sought to evaluate whether the *ES8Z* promoter can be used to transcribe a tRNA-based multiplex sgRNA array in Carrizo citrange. For this to be a viable strategy it needs to at least perform similarly in terms of editing to individual sgRNA cassettes transcribed by the well-established *AtU6-26* promoter (Fig. 2A) [63]. In creating the binary vectors we designed sgRNA targeting four genes in Carrizo citrange (array YCA00039, see Supplementary Table S3) which were used for both sgRNA strategies (Fig. 2A). Aside from the sgRNA expression cassette, all other gene cassettes present on T-DNA of these vectors were comparable (Fig. 2A).

To compare the two strategies we pooled tissue from plants transformed with these vectors. The pools included tissue from 89 plants in the case of the individual sgRNA cassettes driven by *AtU6-26* (*YAO*::*Cas9*_YCV00070)*,* and 46 plants for the *ES8Z* tRNA-based sgRNA array strategy (*YAO*::*Cas9*_ES8Z_YCV00039). We then performed whole genome sequencing on these samples and aligned the reads against a diploid Carrizo citrange genome. For each genomic site targeted by a sgRNA the proportion of edited reads which cause a frameshift at each target allele were assigned into bins ranging from 1 to 10 (where 1 is 0–10% editing 10 is 90–100% editing). The values of these bins were then averaged to get an editing score representative of the activity of a given sgRNA. In this case, these scores were then averaged for each sgRNA present on the vector to obtain a measure of sgRNA activity in the whole population of transgenic plants. Alignment and genotyping statistics are presented in Supplementary Tables S5–S7.

From this analysis we observed similar editing efficiencies for both multiplex editing strategies with a score of 4.6 (s.d. = 1.11) when all sgRNAs were driven by *AtU6-26* in individual sgRNA cassettes, and 5 (s.d. = 2.35) for the *ES8Z* tRNA-based sgRNA array strategy (Fig. 2B). This demonstrates the *ES8Z* promoter is capable of driving the expression of a tRNA-based sgRNA array and that comparable levels of editing can be achieved using either strategy. Nevertheless, there is also potential to increase the editing efficiency in Carrizo citrange.

### Testing of regulatory components for tRNA-based multiplex gene editing in *Citrus*

**Fig. 3:**
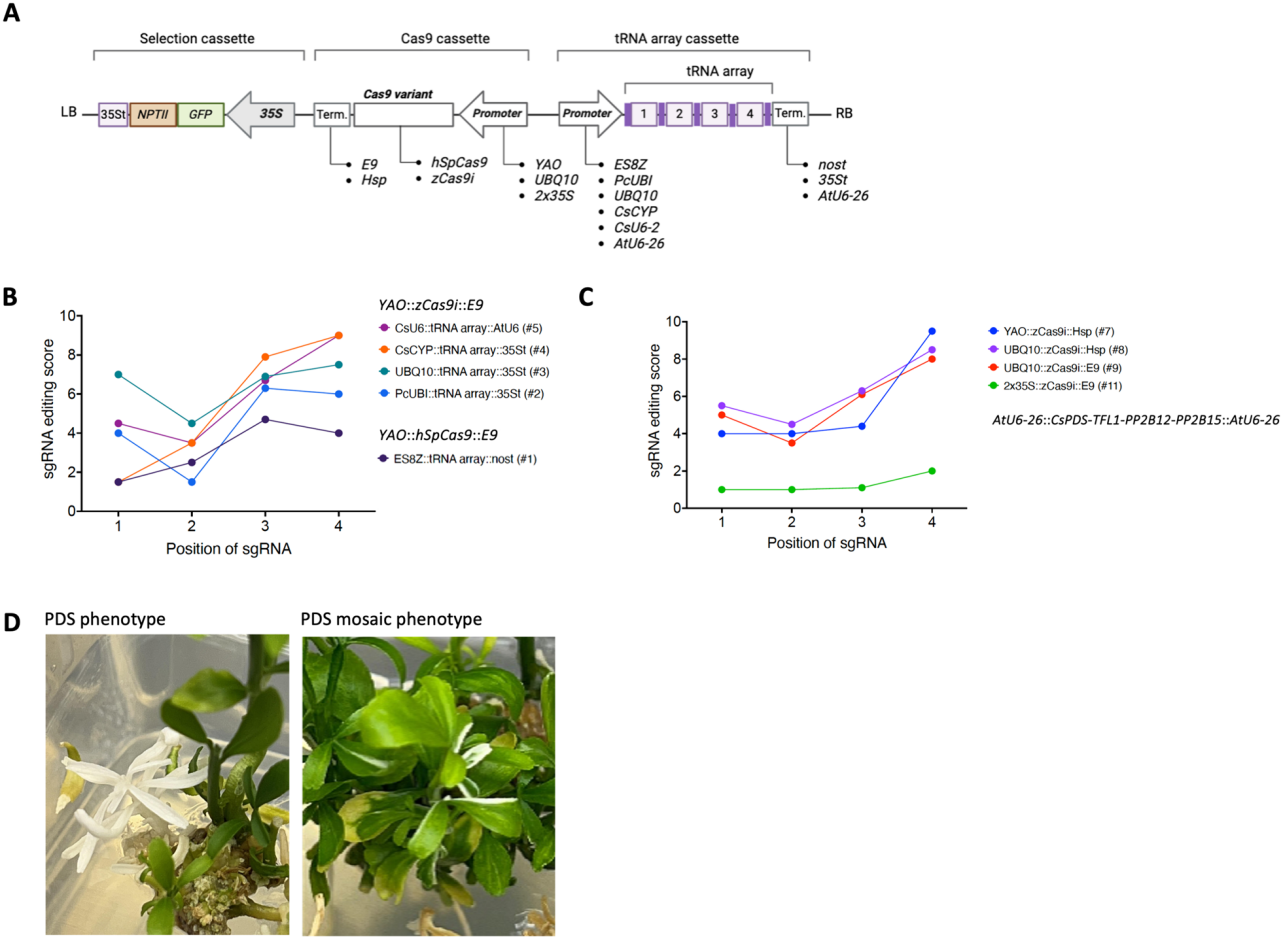
The effect of different regulatory components on the efficiency of multiplex gene editing in *Citrus* using tRNA-based sgRNA arrays. **A** Diagram of the T-DNA architecture with where the promoter regulating the tRNA-based sgRNA array. **B** Variation in gene editing scores (1—low; 10—high) for each sgRNA array promoter tested while a *YAO*::*zCas9i*::*E9* cassette was kept constant. There was an exception in the case of vector #1 which had a *YAO*::*hSpCas9*::*E9* cassette. Scores are ordered by the sgRNA position on the array. **C** Variation in gene editing scores for each zCas9i promoter and terminator combination tested while *AtU6-26 was used to* drive the tRNA array. **D** Shoots of Carrizo citrange *pds* mutant as a result of editing and mosaic phenotype observed in *PDS*-edited Carrizo citrange plants

To determine if the tRNA-based multiplex strategy could be optimized for generating higher editing levels in *Citrus*, we tested different promoters and transcriptional terminators, using the *zCas9i* intron containing *Cas9* variant (Fig. 3A) [15]. These vectors were all constructed using the same backbone vector containing the selection cassette (2 × *35S*::*GFP-NPTII*) we had previously defined [62]. In addition, we included the pAt*YAO*::*Cas9_ES8Z* vector described in the previous section. To compare these components we designed a tRNA-based sgRNA array targeting a new set of four genes (array PDS_TFL1_PP2B12_PP2B15; see Supplementary Table S3), including *PHYTOENE DESATURASE* (*CsPDS*). Targeting *CsPDS* allowed us to have a visual phenotype of editing efficiency as loss of function causes white shoots or mosaic tissue pigmentation (Fig. 3D) in the transformed plants.

We successfully recovered transformed Carrizo citrange plants for nine out of the 11 vectors designed for this experiment, which resulted in a total of 533 plants (Table 2). Transformation efficiencies were over 100% for more than half of the tested vectors, as assessed by GFP + signal in regenerated shoots. This highly successful transformation process is possible because individual explants can yield multiple individual transgenic shoots. Only two vectors (#6 and #10) failed to generate GFP + plants.

**Table 2:** The constituent components of tested CRISPR/Cas9 vectors for improving multiplex gene editing using a tRNA-based sgRNA array

#	Promoter Cas9	Cas9 variant​	Terminator Cas9​	Promoter tRNA array​	tRNA array​	Terminator tRNA array​	#explants​	Transformation efficiency (%)
1	*YAO​*	*hSpCas9​*	*E9​*	*ES8Z*	*CsPDS-TFL1-PP2B12-PP2B15​*	*nost​*	79​	181
2	*YAO​*	*zCas9i​*	*E9​*	*PcUBI​*	*CsPDS-TFL1-PP2B12-PP2B15​*	*35St​*	48​	133
3	*YAO​*	*zCas9i​*	*E9​*	*UBQ10​*	*CsPDS-TFL1-PP2B12-PP2B15​*	*35St​*	66​	52
4	*YAO​*	*zCas9i​*	*E9​*	*CsCYP​*	*CsPDS-TFL1-PP2B12-PP2B15​*	*35St​*	79​	111
5	*YAO​*	*zCas9i​*	*E9​*	*CsU6-2​*	*CsPDS-TFL1-PP2B12-PP2B15​*	*AtU6​-26*	42​	145
6	*YAO​*	*zCas9i​*	*E9​*	*AtU6​-26*	*CsPDS-TFL1-PP2B12-PP2B15​*	*AtU6-26​*	77​	1
7	*YAO​*	*zCas9i​*	*Hsp​*	*AtU6​-26*	*CsPDS-TFL1-PP2B12-PP2B15​*	*AtU6​-26*	64​	106
8	*UBQ10​*	*zCas9i​*	*Hsp​*	*AtU6​-26*	*CsPDS-TFL1-PP2B12-PP2B15​*	*AtU6​-26*	55​	47
9	*UBQ10​*	*zCas9i​*	*E9​*	*AtU6​-26*	*CsPDS-TFL1-PP2B12-PP2B15​*	*AtU6​-26*	39​	105
10	2 × *35S​*	*zCas9i​*	*Hsp​*	*AtU6​-26*	*CsPDS-TFL1-PP2B12-PP2B15​*	*AtU6​-26*	65​	0
11	2 × *35S​*	*zCas9i​*	*E9​*	*AtU6​-26*	*CsPDS-TFL1-PP2B12-PP2B15​*	*AtU6​-26*	66​	11

The explant number and transformation efficiency are also listed

For the five vectors which differed by the components of the tRNA-based sgRNA array cassette (#1–#5), we conducted whole genome sequencing from pooled tissue (Fig. 3A). As above, we calculated an editing score for each sgRNA (Fig. 3B) which varied both by sgRNA and promoter driving the tRNA-based sgRNA array. The constitutive *UBQ10* (At4g05320) promoter [14] from *Arabidopsis* performed best overall, resulting in consistently high editing scores for all four sgRNA (scores of 4.5–7.5). The use of the *CsU6-2* promoter [19] also produced high levels of editing across the sgRNAs. The *CsCYP* and *PcUBI* promoters [9, 10] were more variable with both producing high editing scores for positions 3 and 4, but having very low scores (both 1.5) for positions 1 and 2, respectively. Notably, these four vectors all outperformed the p*AtYAO::Cas9_ES8Z* vector which had a top score of 4.5 for sgRNA 3.

We also tested varying the promoters and terminators regulating *zCas9i*, while maintaining a consistent tRNA-based sgRNA array under the control of the *AtU6-26* promoter (Fig. 3C). Given the results just presented in Fig. 3B, it was surprising to see the variation in editing scores between sgRNA 1–3 to be quite modest, with sgRNA 4 being the highest. In the case of the two vectors featuring the *UBQ10* promoter, as well as the one including the *YAO* promoter, these scores for sgRNA 4 were all at least 8. This demonstrates that the *AtU6-26* promoter is an excellent promoter to drive the tRNA-based sgRNA array. The two *UBQ10* vectors demonstrate that equivalent results are achieved regardless of whether the *E9* or *Hsp18.2* terminator is used, indicating both to be effective. It is also interesting to see similar results from the use of the *YAO* promoter and the *UBQ10* promoter, thus suggesting there are more efficiency gains to be made in optimizing sgRNA expression than the expression of *Cas9*. In this experiment, the 2 × *35S* promoter was the least effective with scores below 2 for all positions of sgRNAs (Fig. 3C).

**Fig. 4:**
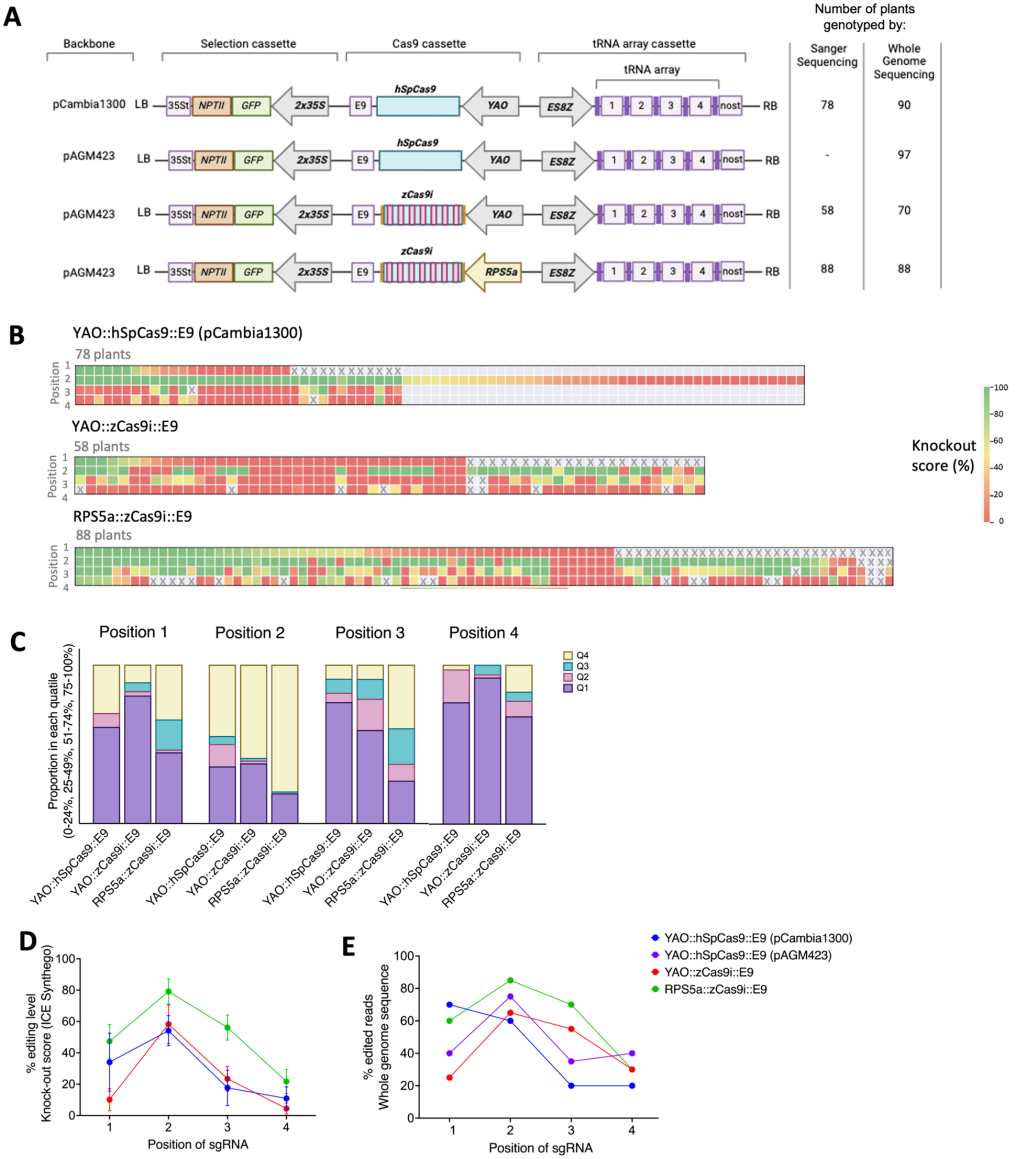
*RPS5a* driving zCas9i increases gene editing efficiency in a multiplex system in Carrizo citrange. **A** Diagram of the vectors containing an *ES8Z* promoter driving the tRNA cassette with the four sgRNAs and number of plants genotyped for each vector. **B** Heatmaps of the knockout scores for the targets of each sgRNA in individual plants. Indicated by a colored box. Grey boxes = no data, X = failed sequencing. Knockout scores are the percentage of sequence likely to disrupt a CDS reading frame estimated by the ICE Analysis tool (v3.0) (Synthego). **C** Proportion of individual plants in a population being classified into quartiles by knockout score, for each of the four sgRNA. **D** Average knock-out scores (ICE Synthego score) of Sanger sequencing genotyping showing variation among sequenced samples (78, 58, and 88 plants) for each position of sgRNA for the lines shown in panel (**B**). **E** Editing scores of the pooled whole genome sequencing of each sgRNA for all tested vectors

In an independent experiment, we compared the performance of *hSpCas9* and *zCas9i* directly using the *YAO* promoter. We tested the *RPS5a* (At3g11940) promoter from *Arabidopsis* [55], to drive *zCas9i*. In *Arabidopsis*, the use of the *RPS5a* promoter to drive *zCas9i* has been shown to be a successful strategy [15]. Given that we have demonstrated that *ES8Z*, another ribosomal gene promoter from *Arabidopsis*, functions well in *Citrus,* we also tested the *RPS5a* promoter here. In this experiment, we used a *ES8Z*-driven tRNA-based sgRNA array cassette with four sgRNAs (array YCA00010; see Supplementary Table S3), different to those previously tested (Fig. 4A).

We initially quantified editing in individual plants for each sgRNA using Sanger sequencing of PCR amplicons. This strategy utilizes primers which amplify both alleles at once. Levels of disruptive editing were then quantified using the Synthego ICE Analysis tool (v3.0) [5]. The knockout scores, which represent the percentage of sequence likely to disrupt a CDS reading frame, are presented in a heatmap in Fig. 4B. The sgRNA at position 2 showed a higher editing efficiency as compared to the other sgRNAs in this cassette. Remarkably, 50% of genotyped plants transformed with a T-DNA containing the *YAO*::*hSpCas9*::*E9* cassette had disruptive gene editing (> 80%) at the sites targeted by this sgRNA. This number modestly increased to 59% when the *YAO*::*zCas9i*::*E9* cassette was used, and substantially increased to 80% with the *RPS5a*::*zCas9i*::*E9* cassette (Fig. 4B).

Strong editing at the targets of the position 2 sgRNA did not necessarily correspond to strong editing at the targets of other sgRNA array positions. We further analyzed plants with (1) genotyping data for at least three of the four sgRNAs and (2) disruptive editing at the targets of position 2 (since in the *YAO*::*hSpCas9*::*E9* population only these plants were genotyped for multiple positions). Strikingly, only 30% of these plants had editing levels above 80% at the targets of another sgRNA in the *YAO*::*hSpCas9*::*E9* population (34 plants). Furthermore, only 13% of plants in the *YAO*::*zCas9i*::*E9* population (30 plants) had disruptive editing at the targets of an additional sgRNA position. In both these cases, it was the targets of sgRNA at position 1 which were most commonly edited. In contrast, in the equivalent plants in the *RPS5a*::*zCas9i*::*E9* population (61 plants), 40% had disruptive editing levels at an additional sgRNA position, a further 15% had such edits for three sgRNA, and 5% had editing for all four sgRNAs in the array (Fig. 4B). These results demonstrate that the use of the *RPS5a*::*zCas9i*::*E9* cassette increases the frequency of editing among T_0_ plants considerably, as well as the successful editing of multiple targets in Carrizo citrange plants.

This conclusion is supported when we consider editing of the transgenic plants in aggregate. When we bin edit levels of the sgRNA targets into quantiles, an increase in the proportion of plants having editing levels in the upper quartiles increases substantially in the *RPS5a*::*zCas9i*::*E9* plants (Fig. 4C). Likewise when we plot the average of the editing scores, the *YAO*::*hSpCas9*::*E9* and *YAO*::*zCas9i*::*E9* populations appear similar, while the *RPS5a*::*zCas9i*::*E9* plants have increased editing across all four sgRNA positions (Fig. 4D). We later performed pooled whole genome sequencing of these populations, with all samples including tissue from over 80 individual plants. We also included a second population of *YAO*::*hSpCas9*::*E9* plants (Fig. 4E). In large part, the resulting editing scores indicated similar levels of editing to what was observed in the averaged Sanger sequencing editing values. Admittedly, there are minor discrepancies between the two genotyping strategies when comparing equivalent constructs (Fig. 4D, E). However, these are likely explained by the larger number of plants included in the pooled whole genome sequencing approach. We conclude that this comparison validates the pooled whole genome sequencing approach in producing accurate estimates of the level of editing within a population.

This series of experiments validates several promoters that can be successfully used in gene editing in *Citrus*, specifically in the context of multiplexing sgRNAs in tRNA-based arrays. The first experiment also demonstrates that zCas9i also improves editing, although this effect is not as obvious in the second experiment.

## Discussion

Gene editing of multiple loci at once using CRISPR/Cas9 requires highly efficient rates of editing. This is all the more necessary in species such as *Citrus* for which transgenic plants are slow and laborious to generate. Here we document our ongoing improvements in implementing multiplex gene editing in the *Citrus* cultivar Carrizo citrange using tRNA-based sgRNA arrays.

We have demonstrated that the *ES8Z* promoter from Arabidopsis is an effective and useful promoter in the generation of transgenic *Citrus*. It is capable of robust expression both in Valencia sweet orange and in Carrizo citrange. Subsequently, we utilized the *ES8Z* promoter to express a tRNA-based sgRNA array to implement multiplex gene editing. With this strategy we observed editing at all targeted genomic loci indicating that this system functions well in Carrizo citrange, consistent with other groups who have reported the use of tRNA-based arrays in *Citrus* [20]. In our hands this system performed similarly well to the use of individual sgRNA each driven by the *AtU6-26* promoter. While encouraging, we judged there was significant room for improvement.

We thus trialed many different variations of CRISPR/*Cas9* vectors. We found that use of the *zCas9i* endonuclease variant, which contains introns, can increase the rate of editing. This is consistent with several other studies [15, 18, 34, 43, 45]. We show, in Fig. 3B, that all transgenes containing the *zCas9i* variant outperformed the vector with *hSpCas9*. While these transgenes all had different promoters expressing the sgRNA arrays, we draw the reader's attention to Fig. 3C where the *YAO*::*zCas9i*::*E9* cassette performs similarly to those which use the *UBQ10* promoter. While not a direct comparison, these results suggest that in Fig. 4B it is the *hSpCas9*, not the *YAO* promoter, which is responsible for the relatively lower levels of editing exhibited by the *YAO::hSpCas9::nost* construct. Having said this, the *zCas9i* variant alone did not make a substantial difference in the experiment presented in Fig. 4. Here the vectors with *YAO*::*hSpCas9*::*E9* and *YAO:*:*zCas9i*::*E9* cassettes performed similarly and it was only when the *zCas9i* variant was paired with the *RPS5a* promoter did significant improvements in editing occur. Nevertheless, we believe that the *zCas9i* variant is the superior one for gene editing in *Citrus*.

Several insights can be drawn from our results concerning tRNA-based sgRNA arrays. First, it is clear across our experiments that the variation in sgRNA editing efficiency is greater than any position-specific effect that using the tRNA-based array may impose. In the experiments depicted in Fig. 4 editing was lowest at the loci targeted by the sgRNA at position 2 of the array, and highest at position 4. For the array in the experiment in Fig. 5 the converse is true, This implies that the order sgRNA are placed in the array has little influence on their function relative to other factors. Second, as is seen in Fig. 4B, some sgRNAs are considerably more efficient at editing than others, in this case it was the sgRNA at position 2. This underscores the need to test multiple sgRNA when comparing CRISPR/*Cas9* vectors, especially when multiplex gene editing is the goal. A single target site cannot be representative of the activity of a strategy. Third, again considering Fig. 4B, it is clear that the degree of editing is not independent for each sgRNA in the array, as in this experiment we rarely observed editing at multiple positions without strong editing at the position 2 targets. This implies it is more likely to obtain plants with multiple edits than plants with well-edited single distinct targets.

In light of the variation in sgRNA editing efficiency, a practical lesson we learned from these experiments was that there is much more to gain by optimizing sgRNA expression than that of *Cas9*. While robust expression of *Cas9* is important, we observed much more variation in editing scores when altering the promoter used to express the sgRNA arrays (Fig. 3B) than altering the promoter used to express *Cas9* (Fig. 3C). Thus, if other researchers were to conduct similar experiments on another species, we suggest that they focus their time and efforts on selecting the promoter driving the sgRNA array.

It was also interesting to observe that while our best editing results when testing different sgRNA array promoters was the Pol II *UBQ10* promoter, we also achieved good results using the Pol III *AtU6-26* and *CsU6-2* promoters. Arrays of sgRNAs are commonly regulated by strong Pol II promoters as these promoters are usually better characterized, especially in non-model species. Our results here suggest that Pol lII promoters can also work very well in this context.

On the subject of well-characterized promoters, in both the expression of the *RUBY* transgene and the expression of *zCas9i*, we observed low activity from the *2X35S* promoter. We know this promoter is active in *Citrus* as we use it to drive our selection cassette. We believe the inclusion of this promoter driving additional transgenes created a duplicated sequence on the T-DNA and likely triggered transgene silencing in these plants, severely reducing their activity [29, 32, 49]. As such, the identification of multiple strong promoters able to drive the different cassettes in a multi-cassette T-DNA is an important element in developing effective vectors. In conducting these experiments several methodological advances were made beyond vector optimization. For example, we quantified the betalain produced by the *ES8Z*::*Ruby*::*nost* transgene as a measure of *ES8Z* activity. While the use of this tripartite gene has quickly become popular, to our knowledge quantification of the pigment produced as a quantification of promoter activity had not been previously reported. Naturally, this is a somewhat coarse measurement, without normalization for tissue input. It is nevertheless a rapid, and potentially useful, screening method which may have other applications. We also developed a pooled whole-genome sequencing pipeline to assay levels of editing in a population of transgenic plants. We later validated this approach by comparison to a sample from the same population genotyped using Sanger sequencing. This is both a labor and cost-efficient approach that does not require additional resources or assays with increasing numbers of loci targeted in multiplex gene editing. This is especially true for heterozygous genomes like those of many *Citrus* cultivars which require allele-specific genotyping.

Admittedly, our testing of promoters was not exhaustive and there are further comparisons which could be made. For instance, it would be worthwhile to compare the *UBQ10*::*zCas9i*::*E9* and the *RPS5a*::*zCas9i*::*E9* cassettes directly using a common array. It would also be informative to test the *RPS5a*::*zCas9i*::*E9* with different tRNA-based sgRNA array promoters. We can also not discount that other promoters we have not tried may improve editing rates further still. Additionally, we do not know the current limits of expression of tRNA-based sgRNA arrays. Here we tested four sgRNAs in each tested array, whether this can be expanded further and what this would cost in terms of editing efficiency is currently unknown.

Ultimately, we have compared a range of promoters for their ability to effectively edit multiple loci using CRISPR/*Cas9* via a tRNA-based sgRNA array strategy. Some of the promoters we have worked with have previously been shown to be efficient in CRISPR/Cas9 editing in *Citrus* (i.e. *CsU6-2* and *RPS5a*) [19, 55], however, the efficiency of editing multiple loci simultaneously has not previously been explored. Furthermore, we show the effectiveness of several promoters which have not before been shown to be active in *Citrus* (e.g. *UBQ10*, *ES8Z*).

This study has resulted in substantial efficiency gains such that editing multiple genes in Carrizo citrange simultaneously is a realistic proposition in an individual experiment. We find that use of the *zCas9i* endonuclease variant with either the *UBQ10* or *RPS5a* promoter is a solid strategy for multiplex gene editing in *Citrus* when coupled with a promoter such as *AtU6-26*, *CsU6-2* or *ES8Z* expressing a tRNA-based sgRNA array. These improvements should be of broad utility to researchers conducting such experiments in *Citrus,* or potentially other non-model eudicot plant species.

## Supplementary Information

**Supplementary Tables S1–S11: Table S1**: Plasmids - A list of all the plasmids generated and used in this study. **Table S2:** Plasmid Assembly - The component plasmids used to construct binary vectors using Golden Gate Cloning. **Table S3:** Gene Models - Gene models homologous to the sequences targeted in this study. **Table S4:** Primers - The primers used in this study. **Table S5:** WGS alignment statistics - Alignment statistics from pooled whole genome sequencing data. **Table S6:** WGS Results - Allele specific genotyping results from pooled whole genome sequencing data. **Table S7:** WGS Results Summary - Summary statistics of genotyping results for each vector from pooled whole genome sequencing data. **Table S8:** WGS data -  List of pooled whole genome sequencing datasets. **Table S9:** PDS phenotype incidence - Frequency of an observed PDS phenotype in plants transformed with the PDS_TFL1_PP2B12_PP2B15 array. **Table S10:** Sanger Summary - Summary of Sanger sequencing results for vectors presented in Figure 4B. **Table S11:** Sanger KO Scores - Synthego Knockout scores for the Sanger sequencing results for vectors presented in Figure 4B. **Supplemental Fig S1:** Characterization of *RUBY* expression in Carrizo citrange shows variation in expression and decrease over time. **A** Variation of *RUBY* expression among transformed plants. Each leaf is from an independent transgenic plant at approximately the same developmental stage. **B** Betalain concentration decreases in leaves over time. **C** New leaves show higher expression. **D** Roots showing high expression of *RUBY*.

**Supplementary File 1:** arrays.fa—Sequence of tRNA-based sgRNA arrays used in this study. **Supplementary File 2:** targets.fa—Carrizo citrange genomic regions targeted by CRISPR/Cas9 reagents with 1 kilobase of sequence either side.
