# Supplementary figures and images for: An efficient multiplex approach to CRISPR/Cas9 gene editing in citrus
Source: Plant Methods. 2024 Sep 28;20:148. doi: 10.1186/s13007-024-01274-4 (PMC11438372; doi:10.1186/s13007-024-01274-4)

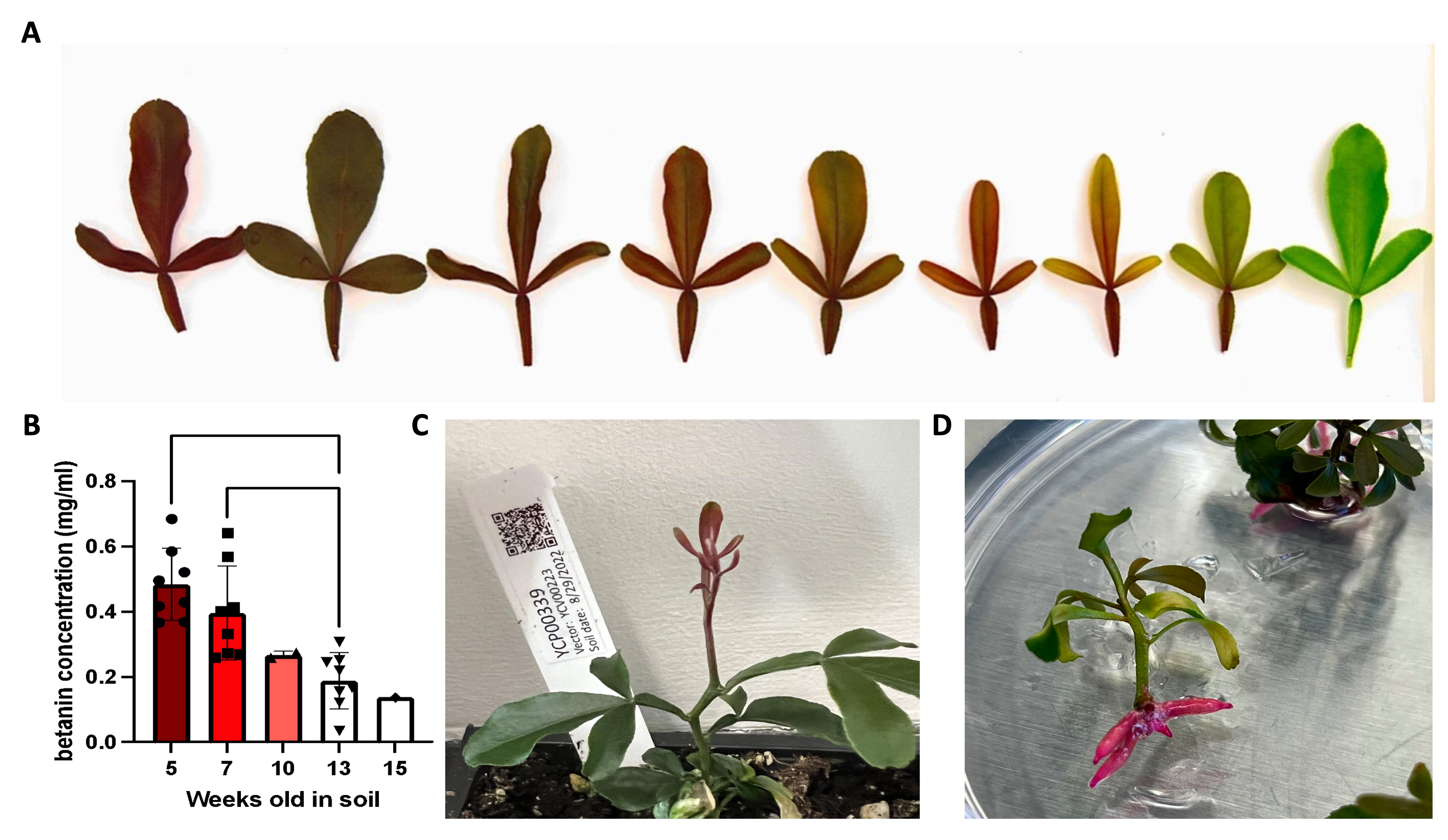

Supplement: Supplementary file 1 — Supplementary Tables S1–S11: Table S1: Plasmids - A list of all the plasmids generated and used in this study. Table S2: Plasmid Assembly - The component plasmids used to construct binary vectors using Golden Gate Cloning. Table S3: Gene Models - Gene models homologous to the sequences targeted in this study. Table S4: Primers - The primers used in this study. Table S5: WGS alignment statistics - Alignment statistics from pooled whole genome sequencing data. Table S6: WGS Results - Allele specific genotyping results from pooled whole genome sequencing data. Table S7: WGS Results Summary - Summary statistics of genotyping results for each vector from pooled whole genome sequencing data. Table S8: WGS data - List of pooled whole genome sequencing datasets. Table S9: PDS phenotype incidence - Frequency of an observed PDS phenotype in plants transformed with the PDS_TFL1_PP2B12_PP2B15 array. Table S10: Sanger Summary - Summary of Sanger sequencing results for vectors presented in Figure 4B. Table S11: Sanger KO Scores - Synthego Knockout scores for the Sanger sequencing results for vectors presented in Figure 4B. Supplemental Fig S1: Characterization of RUBY expression in Carrizo citrange shows variation in expression and decrease over time. A Variation of RUBY expression among transformed plants. Each leaf is from an independent transgenic plant at approximately the same developmental stage. B Betalain concentration decreases in leaves over time. C New leaves show higher expression. D Roots showing high expression of RUBY. [file 13007_2024_1274_MOESM1_ESM.jpg]
